# Anti-deadzone adaptive fuzzy dynamic surface control for planar space robot with elastic base and flexible links

**DOI:** 10.1038/s41598-023-48750-w

**Published:** 2023-12-07

**Authors:** Xiao-qin Huang, Deng-feng Huang

**Affiliations:** 1 College of Physics and Electronic Information Engineering, Minjiang University, Fuzhou City, 350108 Fujian Province People’s Republic of China; 2 School of Mechanical and Automobile Engineering, Fujian University of Technology, Fuzhou City, 350118 Fujian Province People’s Republic of China

**Keywords:** Aerospace engineering, Mechanical engineering

## Abstract

In order to combat the impact of the dead zone and reduce vibration of the space robot's elastic base and flexible links, the trajectory tracking and vibration suppression of a multi-flexible-link free-floating space robot system are addressed. First, the elastic connection between the base and the link is considered as a linear spring. Then the assumed mode approach is used to derive the dynamic model of the flexible system. Secondly, a slow subsystem characterizing the rigid motion and a fast subsystem relating to vibration of the elastic base and multiple flexible links are generated utilizing two-time scale hypotheses of singular perturbation. For the slow subsystem with a dead zone in joint input torque, a dynamic surface control method with adaptive fuzzy approximator is designed. Dynamic surface control scheme is adopted to avoid calculation expansion and to simplify calculation. The fuzzy logic function is applied to approximate uncertain terms of the dynamic equation including the dead zone errors. For the fast subsystem, an optimal linear quadratic regulator controller is used to suppress the vibration of the multiple flexible links and elastic base, ensuring the stability and tracking accuracy of the system. Lastly, the simulation results verify the effectiveness of the proposed control strategy.

## Introduction

Space robots can assist or replace astronauts to complete space on-orbit tasks, and their application can greatly improve the efficiency of human exploration of space^1–5^. Therefore, various space robots have been subjected to dynamic control studies by academics^6–10^. As the requirements for control accuracy are increasing, various flexibilities of space robot become increasingly prominent^11–13^. The space robot itself is light and has long arms, so it will cause flexible vibration during operation. In order to expand the working range of space robots, they are installed on a mobile base on the space station, allowing the base to move along the guide rails assembled by the truss. Space robots and their payloads are usually large, so during the operation process, the elastic vibrations of guide rails are inevitably excited. The coupling relationship between the base and the links, along with the flexible vibrations, causes tracking deviation^14^. Therefore, researchers working in the field of space robot control should not only focus on trajectory tracking, but also take vibration suppression of link flexibility and base elasticity into consideration in order to achieve high precision control^15^. Yu et al.^16^ adopted an adaptive control method to achieve trajectory tracking of multi-flexible-link space robot, and applied an optimal control method to achieve flexible vibration suppression. Lu et al.^17^ proposed the criteria for optimally arranging piezoelectric actuators in flexible manipulators using the particle swarm optimization algorithm. In order to quickly suppress the flexible vibration, Joono et al.^18^ created a direct parameter updating rule and regarded the model uncertainty of the multi-link flexible robot as a parameter perturbation. Aiming at the coordinated motion between the base’s attitude and the joint angle of a flexible space manipulator, Huang et al.^19^ designed a neural network compensation control scheme based on hybrid trajectories that reflects both the flexible mode and the rigid body motion to actively suppress the flexible vibration. To achieve the desired trajectory tracking and to reduce vibration caused by the flexible joint and flexible link, Xie et al.^20^ proposed a robust fuzzy sliding mode control for the flexible link and flexible joint space manipulator system with external interference and uncertain parameters. They also developed a speed difference feedback control and a linear quadratic optimal control to do so. Under the influence of base elasticity, Liang et al.^21^ applied a cascade control approach to counteract the influence on the flexible joint space robot's elastic base and flexible joint while disregarding the flexible vibration of the arm. Without knowing anything about the system model, Fu et al.^22^ designed an input-constrained repetitive learning control algorithm for motion and vibration. It successfully reduced the flexible vibration of the base, the arms, and the joints while realizing high-precision tracking of periodic signals.

Previous studies are mainly based on the ideal condition of complete joint torque. In fact, the deadzone is a common nonlinear characteristic of actuators. The negative effects of the "deadzone" include affecting steady-state tracking error, producing limit cycle oscillation, and even leading to control failure. Consequently, it is necessary to build an appropriate "dead-zone" compensating approach in order to fulfill the high precision control requirements of space robots. To completely minimize the impact of the dead zone on the floating base plane three-link space manipulator system and to guarantee the successful application of tracking control, Zhang et al.^23^ proposed an adaptive control to approach the upper bound of deadzone characteristics. In order to reduce the effect of dead zone on joint input torque, Huang et al.^24^ introduced a hybrid trajectory control method based on a compensator and a recurrent neural network controller. This method increases the precision of trajectory tracking while simultaneously achieving trajectory tracking and vibration suppression. Chen et al.^25^ addressed a distributed controller which aims to solve the master–slave consistency problem of multiple flexible manipulators with undetermined parameters, unidentified disturbances, and actuator dead zone. The suggested controller's convergence along the number of iterations can be accelerated by the dead-time inversion, which can also improve control precision.

The backstepping method in^26^ is an effective method for dynamic control of space robots. However, the problem of "differential explosion" will arise as a result of the repeated derivation of the virtual control component during the backstepping operation. Therefore, Swaroop et al.^27^ put forward the concept of dynamic surface in the research process, designed a first-order low-pass filter to estimate the virtual control component, and solved this issue. Fuzzy logic is indeed a powerful technique for handling nonlinear systems with stochastic behavior and varying inputs^28^. Combined with fuzzy logic system, Park et al.^29^ designed a dynamic surface sliding mode control method with a state observer and a parameter estimator. Aiming at the "complexity explosion" problem in the design of traditional adaptive backstepping controller, Zhou et al.^30^ proposed an adaptivefuzzy backstepping controller based on dynamic surface control, which tackled the effect of external interference and modelling error of the robot. An adaptive neural network dynamic surface control approach was put out by Lin^31^, which simplified designof the controller. Li et al.^32^ used a fuzzy neural network disturbance observer to estimate the model uncertainty and disturbance of air-breathing hypersonic vehicle, and developed an adaptive dynamic surface controller. The dynamic surface control approach developed by Dong^33^ uses a first-order integral filter to determine the derivative of the virtual control, which eliminated the expansion of the differential term and simplified the parameter estimation of the controller. The control of a type of single-link manipulator systems with random disturbance and numerous restrictions was covered by Guo et al. in their study^34^. The power index term was created to help stability analysis and fixed-time control, and enhanced dynamic surface control technology and filtering strategy were also developed. Mohammad et al.^35^ proposed an intelligent variable impedance control method coupled with fuzzy gain dynamic surface to improve the interaction between the robot and the unknown changing environment, so that the end effector of the manipulator could track the expected impedance distribution in the presence of large disturbance.

The challenge addressed in this paper is how to design a controller for a multi-flexible-link FFSR with elastic base, considering the existence of dead zone, so that the system can complete the trajectory tracking and suppress the vibration of the flexible links and elastic base, in order to obtain high-precision tracking performance. The main contributions are as follows:

1. Based on two-time scale assumptions, the system is divided into a slow subsystem representing the rigid motion and a fast subsystem describing the flexible vibrations. For the slow subsystem, a dynamic surface controller with an adaptive fuzzy approximator is designed to tackle dead zone. The application of dynamic surface avoids the calculation expansion caused by backstepping method and reduces the calculation amount.
2. The fuzzy logic function approximates the dynamic uncertainty including dead zone error, so that the desired point to point trajectory tracking of the base’s attitude and joints angle can be achieved.
3. For the fast subsystem, the linear quadratic regulator (LQR) is used to suppress the flexible vibration of the base and links concurrently.

There are six sections in this essay. In section "System dynamics equation", the dynamic equations for a planar space robot with an elastic base and multiple flexible links are determined. In Section "Decomposition of fast and slow systems", based on the singular perturbation method, the system is decomposed into the fast and slow subsystems. Section "Design of combined control law" designs an anti-deadzone adaptive fuzzy dynamic surface control method for the slow subsystem and a linear quadratic controller for the fast subsystem. Section "Simulation experiment" shows the simulation results and analysis comparison of a space robot with an elastic base and two flexible links. Section "Conclusions" gives the conclusion.

## System dynamics equation

**Figure 1:**
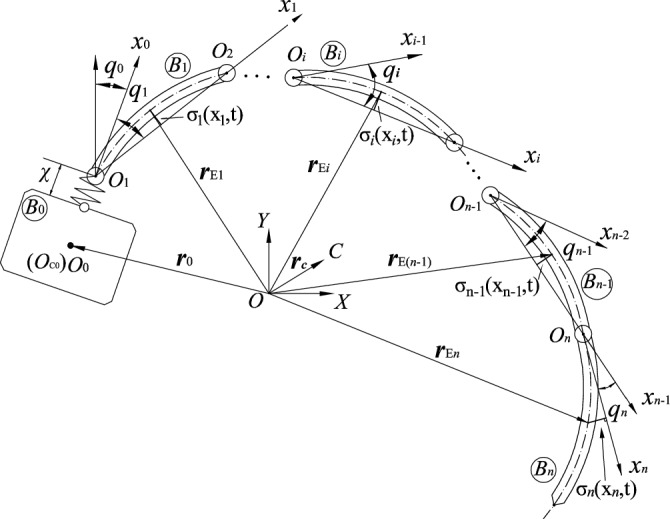
Space Robot System with Elastic base and Flexible Links.

Taking the space robot with elastic base and multi-flexible links as the research object, the system consists of a free-floating base $B_{0}$ and multi-flexible links $B_{i} {(}i = 1,2 \cdots ,n{)}$. The model is shown in Fig. 1. The elasticity of the guide rail is simplified as a light spring to represent the elasticity of the base, and the elastic displacement is denoted as $\chi$. It is assumed that: (1) the spring is a massless spring; (2) The spring only performs the pulling and retracting movement along the axis; (3) Spring elasticity coefficient $k_{\chi }$ is constant; (4) The initial displacement of the spring is zero.

Establish the conjoined coordinate $O_{i} x_{i} y_{i}$ of each split $B_{i} \left( {i = 0,1, \ldots ,n} \right)$, where $O_{0}$ coincides with the centroid $O_{{{\text{c}}0}}$ of $B_{0}$, and $O_{i} \left( {i = 1,2, \ldots ,n} \right)$ is the center of the corresponding rotary hinge. The light spring connects $O_{1}$ and $B_{0}$, the $x_{i} \left( {i = 0, \ldots ,n - 1} \right)$ axis is collinear with $O_{i} O_{i + 1} \left( {i = 0, \ldots ,n - 1} \right)$, and the $x_{n}$ axis and $B_{n}$ are always tangent to $O_{n}$. In the initial state, the distance between $O_{1}$ and $O_{0}$ is $l_{0}$. The length of $B_{i} \left( {i = 1,2, \ldots ,n} \right)$ along the $x_{i}$ axis is $l_{i}$. $C$ is the total centre of mass of the system. The mass of the base and the moment of inertia about the centre of mass are $m_{0}$ and $J_{0}$, respectively. The translational inertial coordinate system $\left( {O - XY} \right)$ is established with any point $O$ in space as the origin.

The links are assumed to be slender and homogeneous, and vibrate transversely in the plane. The bending deformations are mainly considered, and the axial and shear deformations are neglected. The linear density of flexible arm $B_{i} \left( {i = 1,2, \ldots ,n} \right)$ is $\rho_{i} \left( {i = 1,2, \ldots ,n} \right)$, and the bending stiffness of the section is $\left( {EI} \right)_{i}$. According to the theory of vibration mechanics, the flexible links can be thought of as Euler Bernoulli beams, and their elastic deformations can be recorded as:

1 $$\sigma_{i} \left( {X_{i} ,t} \right) = \sum\limits_{j = 1}^{{\kappa_{i} }} {\omega_{ij} } \left( {X_{i} } \right)\xi_{ij} \left( t \right)$$

where $\sigma_{i} \left( {X_{i} ,t} \right)$ is the transverse elastic deformation of $B_{i}$ at section $X_{i} \left( {0 \le X_{i} \le l_{i} } \right)$, $\omega_{ij} \left( {X_{i} } \right)$ is the modal function of the $j$ th order of $B_{i}$, $\xi_{ij} \left( t \right)$ is the modal coordinate corresponding to $\omega_{ij} \left( {X_{i} } \right)$, and $\kappa_{i}$ is the number of truncated terms. In this paper, simply supported beams $B_{i} {(}i = 1,2, \ldots ,n - 1{)}$ and cantilever beams $B_{n}$, respectively, are considered.

As shown in Fig. 1, it can be seen that the vector ${\boldsymbol{r}}_{\delta i} (i = 1,2, \ldots ,n)$ of any point on each flexible link relatives to the origin $O$ of the inertial coordinate system $\left( {{\text{O}} - {\text{XY}}} \right)$ can be expressed as:

2 $${\text{r}}_{\delta i} = {\text{r}}_{0} + \chi {\text{e}}_{x0} + \sum\limits_{j = 0}^{i - 1} {l_{j} {\text{e}}_{xj} } + X_{i} {\text{e}}_{xi} + \sigma_{i} (X_{i} ,t){\text{e}}_{yi}$$

where $$e_{xj} = \left( {\begin{array}{*{20}c} {\cos \left( {\sum\limits_{k = 0}^{j} {q_{k} } } \right)} & {\sin \left( {\sum\limits_{k = 0}^{j} {q_{k} } } \right)} \\ \end{array} } \right)^{T}$$, and $$e_{yj} = \left( {\begin{array}{*{20}c} { - \sin \left( {\sum\limits_{k = 0}^{j} {q_{k} } } \right)} & {\cos \left( {\sum\limits_{k = 0}^{j} {q_{k} } } \right)} \\ \end{array} } \right)^{T}$$$$(j = 0,1, \ldots ,i)$$.

Then according to the total centre of mass theorem of the system

3 $$m_{0} {\boldsymbol{r}}_{0} + \sum\limits_{i = 1}^{n} {\rho_{i} \int_{0}^{{l_{i} }} {{\boldsymbol{r}}_{\delta i} } dX_{i} } = M{\boldsymbol{r}}_{c}$$

where $$m_{0} + \sum\limits_{i = 1}^{n} {\rho_{i} l_{i} } = M$$

${\boldsymbol{r}}_{0}$ and ${\boldsymbol{r}}_{\delta i} \left( {i = 1,2, \ldots ,n} \right)$ are deduced as

4 $${\boldsymbol{r}}_{0} = {\boldsymbol{r}}_{c} + \sum\limits_{j = 0}^{n} {L_{j} } {\text{e}}_{xj} + \sum\limits_{j = 1}^{n} {L_{n + j} } {\text{e}}_{yj}$$

5 $${\boldsymbol{r}}_{\delta i} = {\boldsymbol{r}}_{c} + \sum\limits_{j = 0}^{n} {L_{j} } {\text{e}}_{xj} + \sum\limits_{j = 1}^{n} {L_{n + j} } {\text{e}}_{yj} + \sum\limits_{j = 0}^{i - 1} {l_{j} {\text{e}}_{xj} } + X_{i} {\text{e}}_{xi} + \sigma_{i} (X_{i} ,t){\text{e}}_{yi}$$

where $L_{j} (j = 0,1, \ldots ,2n)$ are the combined functions of the inertia parameters of the system.

Take the derivative of Eq. (5) to time $t$, the velocity vector ${\dot{\boldsymbol{r}}}_{\delta i} (i = 1,2, \ldots ,n)$ can be obtained.

The total kinetic energy ${\text{T}}$ of the space robot system with elastic base and flexible links can be expressed as:

6 $$T{ = }T_{{0}} { + }\sum\limits_{i = 1}^{n} {T_{\delta i} }$$

where $$T_{{0}} { = }\frac{1}{2}\left( {m_{0} {\dot{\text{r}}}_{0}^{2} + J_{0} \dot{q}_{0}^{2} } \right)$$, and $$T_{\delta i} { = }\frac{1}{2}\rho_{i} \int_{0}^{{l_{i} }} {{\dot{\text{r}}}_{\delta i}^{2} } dX_{i} \left( {i = 1,2, \ldots ,n} \right)$$

Because the space robot system is in weightlessness in the outer space environment, the total potential energy $U$ of the space robot system with elastic base and flexible links is derived from the sum of the bending strain energy of the flexible links and the elastic potential energy of the elastic base:

7 $$U = \frac{1}{2}\sum\limits_{i = 1}^{n} {\left( {EI} \right)_{i} \int_{0}^{{l_{i} }} {\left( {\frac{{\partial^{2} \sigma_{i} \left( {X_{i} ,t} \right)}}{{\partial X_{i}^{2} }}} \right)^{2} d} } X_{i} + \frac{1}{2}k_{\chi } \chi^{2}$$

Without losing of generality, assuming that the initial momentum of the system is zero, that is, ${\dot{\boldsymbol{r}}}_{c} = 0$. The dynamic equations of the fully elastic space robot with elastic base and flexible links can be obtained by using the Lagrange equation of the second kind:

8 $${\boldsymbol{M}}\left( {{\boldsymbol{q}},{{\varvec{\updelta}}}} \right)\left[ {\begin{array}{*{20}c} {{\boldsymbol{\ddot{q}}}} \\ {{\boldsymbol{\ddot{\delta }}}} \\ \end{array} } \right] + {\boldsymbol{h}}\left( {{\boldsymbol{q}},{{\varvec{\updelta}}},{\dot{\boldsymbol{q}}},{\dot{\boldsymbol{\delta }}}} \right)\left[ {\begin{array}{*{20}c} {{\dot{\boldsymbol{q}}}} \\ {{\dot{\boldsymbol{\delta }}}} \\ \end{array} } \right] + \left[ {\begin{array}{*{20}c} {\boldsymbol{0}} \\ {{\boldsymbol{K}}_{\xi } {{\varvec{\upxi}}}} \\ {k_{\chi } \chi } \\ \end{array} } \right] = \left[ {\begin{array}{*{20}c} {{\varvec{\uptau}}} \\ {\boldsymbol{0}} \\ \end{array} } \right]$$

where $${\boldsymbol{q}} = \left[ {\begin{array}{*{20}c} {q_{0} } & \cdots & {q_{n} } \\ \end{array} } \right]^{{\text{T}}}$$ is the rigid generalized coordinate column vector of the base’s attitude and the relative rotation angle of the joints of the links,${{\varvec{\upxi}}} = \left[ {\xi_{11} , \ldots ,\xi_{{1\kappa_{1} }} , \ldots ,\xi_{n1} , \ldots ,\xi_{{n\kappa_{n} }} } \right]^{{\text{T}}}$ is the generalized coordinate column vector of the flexible modes of the links, $${{\varvec{\updelta}}} = \left[ {\begin{array}{*{20}c} {{{\varvec{\upxi}}}^{{\text{T}}} } & \chi \\ \end{array} } \right]^{{\text{T}}}$$. ${\boldsymbol{M}}\left( {{\boldsymbol{q}},{{\varvec{\updelta}}}} \right) \in {\boldsymbol{R}}^{{\left( {n + 2 + \sum\limits_{i = 1}^{n} {\kappa_{i} } } \right) \times \left( {n + 2 + \sum\limits_{i = 1}^{n} {\kappa_{i} } } \right)}}$ is a symmetric and positive definite mass matrix.$${\boldsymbol{h}}\left( {{\boldsymbol{q}},{{\varvec{\updelta}}},{\dot{\boldsymbol{q}}},{\dot{\boldsymbol{\delta }}}} \right)\left[ {\begin{array}{*{20}c} {{\dot{\boldsymbol{q}}}^{{\text{T}}} } & {{\dot{\boldsymbol{\delta }}}^{{\text{T}}} } \\ \end{array} } \right]^{{\text{T}}} \in {\boldsymbol{R}}^{{n + 2 + \sum\limits_{i = 1}^{n} {\kappa_{i} } }}$$ is the column vector containing Coriolis force and centrifugal force. ${\boldsymbol{K}}_{\xi } = {\text{diag}}\left( {k_{11} , \ldots ,k_{{1\kappa_{i} }} , \ldots ,k_{n1} , \ldots ,k_{{n\kappa_{n} }} } \right) \in {\boldsymbol{R}}^{{\left( {\sum\limits_{i = 1}^{n} {\kappa_{i} } } \right) \times \left( {\sum\limits_{i = 1}^{n} {\kappa_{i} } } \right)}}$, $k_{ij} = \left( {{\text{EI}}} \right)_{i} \int_{0}^{{l_{i} }} {\omega_{ij}^{{\prime \prime {\text{T}}}} } \omega^{\prime \prime } {\text{d}}x_{i}$ is the stiffness matrix of the links. ${{\varvec{\uptau}}} \in {\boldsymbol{R}}^{n + 1}$ is the torque column vector of the base and links’ joints.

## Decomposition of fast and slow systems

During the operation of the space robot, with the movements of links and base, not only do the multiple flexible links deform and vibrate, but the base also oscillates. The dynamic model is divided into a slow subsystem of rigid motion and a fast subsystem that reflects base elasticity and flexible vibration of links in order to accomplish high-precision control and vibration suppression of space robots. As a result, Eq. (5) is written as a block matrix:

9 $$\left[ {\begin{array}{*{20}c} {{\boldsymbol{M}}_{ss} } & {{\boldsymbol{M}}_{sf} } \\ {{\boldsymbol{M}}_{fs} } & {{\boldsymbol{M}}_{ff} } \\ \end{array} } \right]\left[ {\begin{array}{*{20}c} {{\boldsymbol{\ddot{q}}}} \\ {{\boldsymbol{\ddot{\delta }}}} \\ \end{array} } \right] + \left[ {\begin{array}{*{20}c} {{\boldsymbol{h}}_{ss} } & {{\boldsymbol{h}}_{sf} } \\ {{\boldsymbol{h}}_{fs} } & {{\boldsymbol{h}}_{ff} } \\ \end{array} } \right]\left[ {\begin{array}{*{20}c} {{\dot{\boldsymbol{q}}}} \\ {{\dot{\boldsymbol{\delta }}}} \\ \end{array} } \right] + \left[ {\begin{array}{*{20}c} {\boldsymbol{0}} \\ {{\boldsymbol{K}}_{\xi } {{\varvec{\upxi}}}} \\ {k_{\chi } \chi } \\ \end{array} } \right] = \left[ {\begin{array}{*{20}c} {{\varvec{\uptau}}} \\ {\boldsymbol{0}} \\ \end{array} } \right]$$

where $${\boldsymbol{M}}_{ss}$$, $${\boldsymbol{h}}_{ss} \in {\boldsymbol{R}}^{{\left( {n + 1} \right) \times \left( {n + 1} \right)}}$$. $${\boldsymbol{M}}_{ff}$$, $${\boldsymbol{h}}_{ff} \in {\boldsymbol{R}}^{{\left( {\sum\limits_{i = 1}^{n} {\kappa_{i} } + 1} \right) \times \left( {\sum\limits_{i = 1}^{n} {\kappa_{i} } + 1} \right)}}$$. $$\begin{array}{*{20}c} {{\boldsymbol{M}}_{sf} } \\ \end{array} \begin{array}{*{20}c} = \\ \end{array} \begin{array}{*{20}c} {{\boldsymbol{M}}_{fs}^{{\text{T}}} } \\ \end{array} \in {\boldsymbol{R}}^{{\left( {n + 1} \right) \times \left( {\sum\limits_{i = 1}^{n} {\kappa_{i} } + 1} \right)}}$$. $$h_{sf} \in {\boldsymbol{R}}^{{\left( {n + 1} \right) \times \left( {\sum\limits_{i = 1}^{n} {\kappa_{i} } + 1} \right)}}$$, $$h_{fs} \in {\boldsymbol{R}}^{{\left( {\sum\limits_{i = 1}^{n} {\kappa_{i} } + 1} \right) \times \left( {n + 1} \right)}}$$.

Because ${\boldsymbol{M}}$ is a symmetric and positive definite matrix, its inverse exists:

10 $${\boldsymbol{N}} = {\boldsymbol{M}}^{ - 1} = \left[ {\begin{array}{*{20}c} {{\boldsymbol{N}}_{ss} } & {{\boldsymbol{N}}_{sf} } \\ {{\boldsymbol{N}}_{fs} } & {{\boldsymbol{N}}_{ff} } \\ \end{array} } \right]$$

Defining singular perturbation scale factors $$\varepsilon^{2} = 1/\min \left\{ {k_{11} , \ldots ,k_{{1\kappa_{1} }} , \ldots ,k_{n1} , \ldots ,k_{{n\kappa_{n} }} ,k_{\chi } } \right\}$$,and new variable $${\boldsymbol{z}}$$, $${\tilde{\boldsymbol{K}}}$$$$\left( {{\boldsymbol{z}}\varepsilon^{2} = {{\varvec{\updelta}}},{\tilde{\boldsymbol{K}}} = \varepsilon^{2} {\boldsymbol{K}}} \right)$$. The singular perturbation model of the space robot system with elastic base and flexible links can be obtained from Eq. (9):

11 $${\boldsymbol{\ddot{q}}} = - {(}{\boldsymbol{N}}_{ss} {\boldsymbol{h}}_{ss} + {\boldsymbol{N}}_{sf} {\boldsymbol{h}}_{fs} {)}{\dot{\boldsymbol{q}}} - {(}{\boldsymbol{N}}_{ss} {\boldsymbol{h}}_{sf} + {\boldsymbol{N}}_{sf} {\boldsymbol{h}}_{ff} {)}\varepsilon^{2} {\dot{\boldsymbol{z}}} - {\boldsymbol{N}}_{sf} {\tilde{\boldsymbol{K}}\boldsymbol{z}} + {\boldsymbol{N}}_{ss} {{\varvec{\uptau}}}$$

12 $$\varepsilon^{2} {\boldsymbol{\ddot{z}}} = - {(}{\boldsymbol{N}}_{fs} {\boldsymbol{h}}_{ss} + {\boldsymbol{N}}_{ff} {\boldsymbol{h}}_{fs} {)}{\dot{\boldsymbol{q}}} - {(}{\boldsymbol{N}}_{fs} {\boldsymbol{h}}_{sf} + {\boldsymbol{N}}_{ff} {\boldsymbol{h}}_{ff} {)}\varepsilon^{2} {\dot{\boldsymbol{z}}} - {\boldsymbol{N}}_{ff} {\tilde{\boldsymbol{K}}\boldsymbol{z}} + {\boldsymbol{N}}_{fs} {{\varvec{\uptau}}}$$

According to the singular perturbation model of the system in Eqs. (11) and (12), the following combined control law is designed:

13 $${{\varvec{\uptau}}} = {{\varvec{\uptau}}}_{s} + {{\varvec{\uptau}}}_{f}$$

where ${{\varvec{\uptau}}}_{s}$ is the control torque of the slow subsystem to realize the angle tracking of the base and joints, and ${{\varvec{\uptau}}}_{f}$ is the control torque of the fast subsystem to suppress the vibrations caused by the elastic base and multi-flexible-links at the same time.

In order to deduce the slow subsystem of the space robot with elastic base and flexible links, $\varepsilon$ is set to zero firstly. Then the slowly varying manifold expression $\overline{z}$ of the system can be solved from Eq. (12):

14 $${\overline{\boldsymbol{z}}} = {\tilde{\boldsymbol{K}}}^{ - 1} {\overline{\boldsymbol{N}}}_{ff}^{ - 1} {[} - {(}{\overline{\boldsymbol{N}}}_{fs} {\overline{\boldsymbol{h}}}_{ss} + {\overline{\boldsymbol{N}}}_{ff} {\overline{\boldsymbol{h}}}_{fs} {)}{\dot{\boldsymbol{q}}} + {\overline{\boldsymbol{N}}}_{fs} {{\varvec{\uptau}}}_{s} {]}$$

where the matrix or variable with the dash "￣" means the corresponding slowly varying component.

Substituting the above equation into Eq. (13) and considering ${\overline{\boldsymbol{M}}}_{ss}^{ - 1} = {\overline{\boldsymbol{N}}}_{ss} - {\overline{\boldsymbol{N}}}_{sf} {\overline{\boldsymbol{N}}}_{ff}^{ - 1} {\overline{\boldsymbol{N}}}_{fs}$, the slow subsystem is obtained:

15 $${\overline{\boldsymbol{M}}}_{ss} {\boldsymbol{\ddot{q}}} + {\overline{\boldsymbol{h}}}_{ss} {\dot{\boldsymbol{q}}} = {{\varvec{\uptau}}}_{s}$$

In order to obtain the fast subsystem, defining new variables, and making ${\boldsymbol{p}}_{{1}} = {\boldsymbol{z}} - {\overline{\boldsymbol{z}}}$, ${\boldsymbol{p}}_{2} = \varepsilon {\dot{\boldsymbol{z}}}$, Eq. (12) can be rewritten as:

16 $$\varepsilon {\dot{\boldsymbol{p}}}_{2} = - {(}{\boldsymbol{N}}_{fs} {\boldsymbol{h}}_{ss} + {\boldsymbol{N}}_{ff} {\boldsymbol{h}}_{fs} {)}{\dot{\boldsymbol{q}}} - {(}{\boldsymbol{N}}_{fs} {\boldsymbol{h}}_{sf} + {\boldsymbol{N}}_{ff} {\boldsymbol{h}}_{ff} {)}\varepsilon {\boldsymbol{p}}_{2} - {\boldsymbol{N}}_{ff} {\tilde{\boldsymbol{K}}}{(}{\boldsymbol{p}}_{1} + {\overline{\boldsymbol{z}}}{)} + {\boldsymbol{N}}_{fs} {{\varvec{\uptau}}}$$

Adding the fast variable time scale $\varpi = {t \mathord{\left/ {\vphantom {t \varepsilon }} \right. \kern-0pt} \varepsilon }$, let $\varepsilon = 0$. Then the dynamic equation of fast subsystem is:

17 $$\frac{{{\text{d}}{\boldsymbol{p}}_{{1}} }}{{{\text{d}}\varpi }} = {\boldsymbol{p}}_{2}$$

18 $$\frac{{{\text{d}}{\boldsymbol{p}}_{2} }}{{{\text{d}}\varpi }} = - {\overline{\boldsymbol{N}}}_{ff} {\tilde{\boldsymbol{K}}\boldsymbol{p}}_{1} + {\overline{\boldsymbol{N}}}_{fs} {{\varvec{\uptau}}}_{f}$$

It describes the vibration of the elastic base and flexible links.

## Design of combined control law

### Design of Anti-deadzone adaptive fuzzy dynamic surface controller for slow subsystem

The base position and attitude of the space robot are usually adjusted by momentum wheels or reaction jet devices. The joint hinges are driven by motors, so there is a dead zone in joint input torque.

Considering dead zone in joint input torque, the slow subsystem can be written as:

19 $${\overline{\boldsymbol{M}}}_{ss} {\boldsymbol{\ddot{q}}} + {\overline{\boldsymbol{h}}}_{ss} {\dot{\boldsymbol{q}}} = {{\varvec{\uptau}}}_{s}$$

where $${{\varvec{\uptau}}}_{s} = \left[ {\begin{array}{*{20}c} {\overline{\tau }_{0} } & {D^{{\text{T}}} \left( {{\overline{\boldsymbol{\tau }}}_{r} } \right)} \\ \end{array} } \right]^{{\text{T}}} \in {\boldsymbol{R}}^{n + 1}$$ is the actuator output torque $\overline{\tau }_{0}$ of the base and the joints’ actuator output torque $D\left( {{\overline{\boldsymbol{\tau }}}_{r} } \right) = \left[ {D\left( {\overline{\tau }_{1} } \right) \cdots D\left( {\overline{\tau }_{n} } \right)} \right]^{{\text{T}}}$ of the links in the slow subsystem, and $D\left( {{\overline{\boldsymbol{\tau }}}_{r} } \right)$ is the column vector with the dead zone of the joint input torque ${\overline{\boldsymbol{\tau }}}_{r} = \left( {\overline{\tau }_{1} , \ldots ,\overline{\tau }_{n} } \right)^{{\text{T}}}$.

Since it is typically challenging to acquire the exact parameters of the system dynamics model, let ${{\varvec{\upupsilon}}}_{1} = {\boldsymbol{q}}$, ${\dot{\boldsymbol{\upsilon }}}_{1} = {{\varvec{\upupsilon}}}_{2} = {\dot{\boldsymbol{q}}}$. Then, the slow subsystem can be written as state equation in the following form:

20 $$\left\{ {\begin{array}{*{20}l} {{\dot{\boldsymbol{\upsilon }}}_{1} = {{\varvec{\upupsilon}}}_{2} \begin{array}{*{20}l} {} \\ \end{array} \begin{array}{*{20}l} {} \\ \end{array} \begin{array}{*{20}l} {} \\ \end{array} \begin{array}{*{20}l} {} \\ \end{array} \begin{array}{*{20}l} {} \\ \end{array} \begin{array}{*{20}l} {} \\ \end{array} \begin{array}{*{20}l} {} \\ \end{array} \begin{array}{*{20}l} {} \\ \end{array} \begin{array}{*{20}l} {} \\ \end{array} \begin{array}{*{20}l} {} \\ \end{array} \begin{array}{*{20}l} {} \\ \end{array} \begin{array}{*{20}l} {} \\ \end{array} \begin{array}{*{20}l} {} \\ \end{array} \begin{array}{*{20}l} {} \\ \end{array} \begin{array}{*{20}l} {} \\ \end{array} \begin{array}{*{20}l} {} \\ \end{array} \begin{array}{*{20}l} {} \\ \end{array} \begin{array}{*{20}l} {} \\ \end{array} \begin{array}{*{20}l} {} \\ \end{array} \begin{array}{*{20}l} {} \\ \end{array} \begin{array}{*{20}l} {} \\ \end{array} \begin{array}{*{20}l} {} \\ \end{array} \begin{array}{*{20}l} {} \\ \end{array} \begin{array}{*{20}l} {} \\ \end{array} \begin{array}{*{20}l} {} \\ \end{array} } \\ {{\dot{\boldsymbol{\upsilon }}}_{2} = {\hat{\overline{\boldsymbol{M}}}}_{ss}^{ - 1} \left( {{{\varvec{\upupsilon}}}_{1} } \right){{\varvec{\uptau}}}_{s} - {\boldsymbol{F}}\left( {{{\varvec{\upupsilon}}}_{1} ,{{\varvec{\upupsilon}}}_{2} } \right) + {\hat{\overline{\boldsymbol{M}}}}_{ss}^{ - 1} \left( {{{\varvec{\upupsilon}}}_{1} } \right){\boldsymbol{T}}_{{\Delta }} } \\ \end{array} } \right.$$

where ${\hat{\overline{\boldsymbol{M}}}}_{ss}$, ${\hat{\overline{\boldsymbol{H}}}}_{ss}$ are the nominal models, $\Delta {\boldsymbol{M}} = {\overline{\boldsymbol{M}}}_{ss} - {\hat{\overline{\boldsymbol{M}}}}_{ss}$, $\Delta {\boldsymbol{h}} = {\overline{\boldsymbol{h}}}_{ss} - {\hat{\overline{\boldsymbol{h}}}}_{ss}$, and ${\boldsymbol{F}}\left( {{{\varvec{\upupsilon}}}_{1} ,{{\varvec{\upupsilon}}}_{2} } \right) = {\hat{\overline{\boldsymbol{M}}}}_{ss}^{ - 1} \left( {{{\varvec{\upupsilon}}}_{1} } \right){\hat{\overline{\boldsymbol{h}}}}_{ss} {(}{{\varvec{\upupsilon}}}_{1} ,{{\varvec{\upupsilon}}}_{2} {)}{{\varvec{\upupsilon}}}_{2}$. ${\boldsymbol{T}}_{{\Delta }} = - \Delta {\boldsymbol{M}}{(}{{\varvec{\upupsilon}}}_{1} {)}{\dot{\boldsymbol{\upsilon }}}_{2} - \Delta {\boldsymbol{h}}{(}{{\varvec{\upupsilon}}}_{1} ,{{\varvec{\upupsilon}}}_{2} {)}{{\varvec{\upupsilon}}}_{2}$ is the sum of uncertainties. $\left\| {\Delta {\boldsymbol{M}}} \right\| \le \rho_{{\text{M}}}$, $\left\| {\Delta {\boldsymbol{h}}} \right\| \le \rho_{{\text{h}}}$, $\rho_{i}$($i = {\text{M}},{\text{h}}$) is the normal number.

#### Dead zone in joint input torque

"Dead zone" refers to the range where change in input has no effect on output. It reflects the input–output relationship of zero output after the input of joint torque enters the dead zone. When the signal enters the dead zone, there will be a certain loss, which will result in the deviation of system control.

The joint input torque is ${\overline{\boldsymbol{\tau }}}_{r}$ and the joints’ actuator output torque is $D\left( {{\overline{\boldsymbol{\tau }}}_{r} } \right)$. The simplified dead zone model can be expressed as^36^:

21 $$D\left( {\tau_{i} } \right) = \left\{ {\begin{array}{*{20}l} {m_{ri} \left( {\tau_{i} - b_{ri} } \right)\begin{array}{*{20}l} {} & {} & {} & {\tau_{i} \ge b_{ri} } \\ \end{array} } \\ {0\begin{array}{*{20}l} {} \\ \end{array} \begin{array}{*{20}l} {} \\ \end{array} \begin{array}{*{20}l} {\begin{array}{*{20}l} {\begin{array}{*{20}l} {} \\ \end{array} } \\ \end{array} } \\ \end{array} \begin{array}{*{20}l} {} & {} & {} & {b_{li} < \tau_{di} < b_{ri} } \\ \end{array} } \\ {m_{li} \left( {\tau_{i} - b_{li} } \right)\begin{array}{*{20}l} {} & {} & {} & {\tau_{i} \le b_{li} } \\ \end{array} } \\ \end{array} } \right.$$

where $b_{li} < 0$ and $b_{ri} > 0$ represent the dead zone's left and right relative widths. $m_{li}$ and $m_{ri}$ represent the left and right slopes of the dead zone, respectively, $m_{ri}$ and $m_{li}$ are specified to be greater than zero.

The difference between input and output of dead zone is expressed as:

22 $${\boldsymbol{D}}_{{\Delta }} = {\overline{\boldsymbol{\tau }}}_{r} - D\left( {{\overline{\boldsymbol{\tau }}}_{r} } \right)$$

Therefore, Eq. (20) is rewritten as follows:

23 $$\left\{ {\begin{array}{*{20}l} {\dot{\boldsymbol{\upsilon }}}_{1} = {{\varvec{\upupsilon}}}_{2}\\ {{\dot{\boldsymbol{\upsilon }}}_{2} = {\hat{\overline{\boldsymbol{M}}}}_{ss}^{ - 1} \left( {{{\varvec{\upupsilon}}}_{1} } \right){\overline{\boldsymbol{\tau }}}_{{\text{c}}} - {\hat{\overline{\boldsymbol{M}}}}_{ss}^{ - 1} \left( {{{\varvec{\upupsilon}}}_{1} } \right)\left[ {\begin{array}{*{20}c} 0 & {{\boldsymbol{D}}_{{\Delta }}^{{\text{T}}} } \\ \end{array} } \right]^{{\text{T}}} - {\boldsymbol{F}}\left( {{{\varvec{\upupsilon}}}_{1} ,{{\varvec{\upupsilon}}}_{2} } \right) + {\hat{\overline{\boldsymbol{M}}}}_{ss}^{ - 1} \left( {{{\varvec{\upupsilon}}}_{1} } \right){\boldsymbol{T}}_{{\Delta }} } \\ \end{array} } \right.$$

where $${\overline{\boldsymbol{\tau }}}_{{\text{c}}} = \left[ {\begin{array}{*{20}c} {\overline{\tau }_{0} } & {{\overline{\boldsymbol{\tau }}}} \\ \end{array}_{r}^{{\text{T}}} } \right]^{{\text{T}}}$$ is the torque column vector of the base and joints before passing the "dead zone".

#### Controller design

In this study, the dynamic surface control technique is used to create the virtual control variable and control input signal for the space robot's slow component. This information serves as the foundation for the construction of the slow subsystem's control law ${\overline{\boldsymbol{\tau }}}_{{\text{c}}}$, which enables actual trajectory ${\boldsymbol{q}}$ of the space robot's rigid motion to follow the anticipated trajectory ${\boldsymbol{q}}_{{\text{d}}}$ despite having an elastic base and flexible links.

The design steps of dynamic face control are as follows:

*Step 1*: Define the first error surface as:

24 $${\boldsymbol{s}}_{1} = {{\varvec{\upupsilon}}}_{1} - {\boldsymbol{q}}_{{\text{d}}}$$

Design a virtual control variable ${\tilde{\boldsymbol{\upsilon }}}_{2}$:

25 $${\tilde{\boldsymbol{\upsilon }}}_{2} = {\dot{\boldsymbol{q}}}_{{\text{d}}} - c_{1} {\boldsymbol{s}}_{1}$$

where design constant $c_{1} { > }0$.

With ${\tilde{\boldsymbol{\upsilon }}}_{2}$ as the input and ${{\varvec{\upupsilon}}}_{{2{\text{d}}}}$ as the new state variable output, a first-order low-pass filter (LPF) is introduced:

26 $$\eta_{2} {\dot{\boldsymbol{\upsilon }}}_{{2{\text{d}}}} + {{\varvec{\upupsilon}}}_{{2{\text{d}}}} = {\tilde{\boldsymbol{\upsilon }}}_{2} ,\quad {{\varvec{\upupsilon}}}_{{2{\text{d}}}} \left( 0 \right) = {\tilde{\boldsymbol{\upsilon }}}_{2} \left( 0 \right)$$

where the time $\eta_{2} > 0$.

*Step 2*: Define the second dynamic surface in order to create the control law of the slow subsystem:

27 $${\boldsymbol{s}}_{2} = {{\varvec{\upupsilon}}}_{2} - {{\varvec{\upupsilon}}}_{{2{\text{d}}}}$$

Substituting Eq. (23) into the first derivative of Eq. (27), we can obtain:

28 $${\dot{\boldsymbol{s}}}_{2} = {\hat{\overline{\boldsymbol{M}}}}_{ss}^{ - 1} \left( {{{\varvec{\upupsilon}}}_{1} } \right){\overline{\boldsymbol{\tau }}}_{{\text{c}}} - {\hat{\overline{\boldsymbol{M}}}}_{ss}^{ - 1} \left( {{{\varvec{\upupsilon}}}_{1} } \right)\left[ {\begin{array}{*{20}c} 0 & {{\boldsymbol{D}}_{{\Delta }}^{{\text{T}}} } \\ \end{array} } \right]^{{\text{T}}} - {\boldsymbol{F}}\left( {{{\varvec{\upupsilon}}}_{1} ,{{\varvec{\upupsilon}}}_{2} } \right) + {\hat{\overline{\boldsymbol{M}}}}_{ss}^{ - 1} \left( {{{\varvec{\upupsilon}}}_{1} } \right){\boldsymbol{T}}_{{\Delta }} - {\dot{\boldsymbol{\upsilon }}}_{{2{\text{d}}}}$$

In the study, the fuzzy logic system is used to approximate $${{\varvec{\Theta}}}\left( {\boldsymbol{A}} \right) = \left[ {\begin{array}{*{20}c} 0 & { - {\boldsymbol{D}}_{{\Delta }}^{{\text{T}}} } \\ \end{array} } \right]^{{\text{T}}} + {\boldsymbol{T}}_{{\Delta }}$$, whose exact value is unknown.

${{\varvec{\Theta}}}{(}{\boldsymbol{A}}\left| {\boldsymbol{W}} \right.{)}$ is utilized to approximate ${{\varvec{\Theta}}}{(}{\boldsymbol{A}}{)}$, then

29 $${{\varvec{\Theta}}}{(}{\boldsymbol{A}}\left| {\boldsymbol{W}} \right.{)} = {\boldsymbol{W}}^{T} {\boldsymbol{O}}{(}{\boldsymbol{A}}{)}$$

where ${\boldsymbol{W}}$ is the weight matrix, ${\boldsymbol{O}}{(}A{)} = {[}O_{1} , \ldots ,O_{\gamma } {]}^{{\text{T}}}$ is the fuzzy basis vector, and $\gamma$ is the number of rules. ${\boldsymbol{\rm A}} = \left[ {{{\varvec{\upupsilon}}}_{1}^{{\text{T}}} ,{{\varvec{\upupsilon}}}_{2}^{{\text{T}}} ,{\boldsymbol{q}}_{{\text{d}}}^{{\text{T}}} ,{\dot{\boldsymbol{q}}}_{{\text{d}}}^{{\text{T}}} } \right]^{{\text{T}}}$ is the fuzzy basis network input. The fuzzy basis function $O_{k}$ is expressed as:

30 $$O_{k} {(}A_{1} , \ldots A_{4n} {)} = {{\mathop {\Pi }\limits_{j = 1}^{4n} \mu_{{F_{j}^{k} }} {(}A_{j} {)}} \mathord{\left/ {\vphantom {{\mathop {\Pi }\limits_{j = 1}^{4n} \mu_{{F_{j}^{k} }} {(}A_{j} {)}} {\sum\limits_{k = 1}^{\gamma } {\mathop {\begin{array}{*{20}c} {\Pi } \\ \end{array} }\limits_{j = 1}^{4n} \mu_{{F_{j}^{k} }} {(}A_{j} {)}} }}} \right. \kern-0pt} {\sum\limits_{k = 1}^{\gamma } {\mathop {\begin{array}{*{20}c} {\Pi } \\ \end{array} }\limits_{j = 1}^{4n} \mu_{{F_{j}^{k} }} {(}A_{j} {)}} }}$$

Select the Gaussian membership function:

31 $$\mu_{{F_{j}^{k} }} {(}A_{j} {)} = {\text{exp}}\left[ { - {{{(}A_{j} - a_{jk} {)}^{2} } \mathord{\left/ {\vphantom {{{(}A_{j} - a_{jk} {)}^{2} } {2b_{ij}^{2} }}} \right. \kern-0pt} {2b_{ij}^{2} }}} \right]$$

where $a_{jk}$ and $b_{jk}$ represent the Gaussian membership function's center and breadth, respectively.

The optimal value ${\boldsymbol{W}}^{*}$ of ${\boldsymbol{W}}$ is a constant matrix and satisfies

32 $${\boldsymbol{W}}^{*} = \arg \begin{array}{*{20}c} {\mathop {{\text{min}}}\limits_{{{\text{W}} \in {\Omega }_{{\text{W}}} }} } \\ \end{array} \left[ {\mathop {\sup }\limits_{{A \in {\Omega }_{A} }} \left| {{{\varvec{\Theta}}}\left( {{\boldsymbol{A}}\left| {\boldsymbol{W}} \right.} \right) - {{\varvec{\Theta}}}\left( {\boldsymbol{A}} \right)} \right|} \right]$$

where ${\boldsymbol{W}}^{*}$ is bounded, that is, there is a normal number $\rho_{{\text{W}}}$ , which satisfies $\left\| {\boldsymbol{W}} \right\| \le \rho_{{\text{W}}}$.

Then ${{\varvec{\Theta}}}{(}{\boldsymbol{A}}{)}$ is expressed as follows:

33 $${{\varvec{\Theta}}}\left( {\boldsymbol{A}} \right) = {\boldsymbol{W}}^{*T} {\boldsymbol{O}}\left( {\boldsymbol{X}} \right) + {{\varvec{\upmu}}}^{*}$$

where ${{\varvec{\upmu}}}^{*}$ is the approximation error.

The slow subsystem's control rule is made to:

34 $${\overline{\boldsymbol{\tau }}}_{{\text{c}}} = - c_{2} {\boldsymbol{s}}_{2} - {\boldsymbol{s}}_{1} - {\hat{\boldsymbol{W}}}^{T} {\boldsymbol{O}}\left( {\boldsymbol{A}} \right) - {\hat{\boldsymbol{\mu }}} + {\hat{\overline{\boldsymbol{h}}}}_{ss} {(}{{\varvec{\upupsilon}}}_{1} ,{{\varvec{\upupsilon}}}_{2} {)}{{\varvec{\upupsilon}}}_{{2{\text{d}}}} + {\hat{\overline{\boldsymbol{M}}}}_{ss} \left( {{{\varvec{\upupsilon}}}_{1} } \right){\dot{\boldsymbol{\upsilon }}}_{{2{\text{d}}}}$$

where ${\hat{\boldsymbol{W}}}$ and ${\hat{\boldsymbol{\mu }}}$ are the estimated values of ${\boldsymbol{W}}^{{\boldsymbol{*}}}$ and ${{\varvec{\upmu}}}^{*}$ respectively, and $c_{2} > 0$.

The adaptive regulation law of ${\hat{\boldsymbol{W}}}$ and ${\hat{\boldsymbol{\mu }}}$ is designed:

35 $${\dot{\hat{\boldsymbol{W}}}} = {{\varvec{\upbeta}}}_{1} {\boldsymbol{O}}\left( {\boldsymbol{A}} \right){\boldsymbol{s}}_{2}^{{\text{T}}} - \lambda_{1} {\hat{\boldsymbol{W}}}$$

36 $${\dot{\hat{\boldsymbol{\mu }}}} = {{\varvec{\upbeta}}}_{2} {\boldsymbol{s}}_{2} - \lambda_{2} {\hat{\boldsymbol{\mu }}}$$

where ${{\varvec{\upbeta}}}_{1} \in {\boldsymbol{R}}^{h \times h}$, and ${{\varvec{\upbeta}}}_{2} \in {\boldsymbol{R}}^{{\left( {n + 1} \right) \times \left( {n + 1} \right)}}$. $\lambda_{1} > 0$, $\lambda_{2} > 0$ are the adjustment parameters.

#### Stability analysis

The Lyapunov theory is used to analyze the semi global stability of the slow subsystem with a dead zone in joint input torque.

Define the boundary layer error of slow subsystem:

37 $${{\varvec{\upgamma}}}_{2} = {{\varvec{\upupsilon}}}_{{2{\text{d}}}} - {\tilde{\boldsymbol{\upsilon }}}_{2}$$

Substituting the above equation into Eq. (26), we can obtain:

38 $${\dot{\boldsymbol{\upsilon }}}_{{2{\text{d}}}} = {{ - {{\varvec{\upgamma}}}_{2} } \mathord{\left/ {\vphantom {{ - {{\varvec{\upgamma}}}_{2} } {\eta_{2} }}} \right. \kern-0pt} {\eta_{2} }}$$

Thus, the first derivative of ${{\varvec{\upgamma}}}_{2}$ is obtained:

39 $${\dot{\boldsymbol{\gamma }}}_{2} = - {{{{\varvec{\upgamma}}}_{2} } \mathord{\left/ {\vphantom {{{{\varvec{\upgamma}}}_{2} } {\eta_{2} }}} \right. \kern-0pt} {\eta_{2} }} - {\boldsymbol{\ddot{q}}}_{{\text{d}}} + c_{1} {\dot{\boldsymbol{s}}}_{1} = - {{{{\varvec{\upgamma}}}_{2} } \mathord{\left/ {\vphantom {{{{\varvec{\upgamma}}}_{2} } {\eta_{2} }}} \right. \kern-0pt} {\eta_{2} }} + {{\varvec{\Phi}}}\left( {{\boldsymbol{s}}_{1} ,{\boldsymbol{s}}_{2} ,{\boldsymbol{q}}_{{\text{d}}} ,{\dot{\boldsymbol{q}}}_{{\text{d}}} ,{\boldsymbol{\ddot{q}}}_{{\text{d}}} ,{{\varvec{\upgamma}}}_{2} } \right)$$

where ${{\varvec{\Phi}}}\left( {{\boldsymbol{s}}_{1} ,{\boldsymbol{s}}_{2} ,{\boldsymbol{q}}_{{\text{d}}} ,{\dot{\boldsymbol{q}}}_{{\text{d}}} ,{\boldsymbol{\ddot{q}}}_{{\text{d}}} ,{{\varvec{\upgamma}}}_{2} } \right)$ is a nonnegative continuous function matrix.

Using Eqs. (25) – (27) and (37), the first derivative ${\dot{\boldsymbol{s}}}_{1}$ of the first error surface is obtained:

40 $${\dot{\boldsymbol{s}}}_{1} = - c_{1} {\boldsymbol{s}}_{1} + {\boldsymbol{s}}_{2} + {{\varvec{\upgamma}}}_{2}$$

Utilizing Eq. (33) and the control law Eq. (34), substituting them into Eq. (28), ${\dot{\boldsymbol{s}}}_{2}$ is written as follows:

41 $${\dot{\boldsymbol{s}}}_{2} = {\hat{\overline{\boldsymbol{M}}}}_{ss}^{ - 1} \left( {{{\varvec{\upupsilon}}}_{1} } \right)\left\{ { - c_{2} {\boldsymbol{s}}_{2} - {\boldsymbol{s}}_{1} + {\tilde{\boldsymbol{W}}}^{{\text{T}}} {\boldsymbol{O}}\left( {\boldsymbol{A}} \right) + {\tilde{\boldsymbol{\mu }}} - } \right.\left. {{\hat{\overline{\boldsymbol{h}}}}_{ss} {(}{{\varvec{\upupsilon}}}_{1} ,{{\varvec{\upupsilon}}}_{2} {)}{\boldsymbol{s}}_{2} } \right\}$$

where $${\tilde{\boldsymbol{W}}} = {\boldsymbol{W}}^{{\boldsymbol{*}}} - {\hat{\boldsymbol{W}}}$$, and $${\tilde{\boldsymbol{\mu }}} = {{\varvec{\upmu}}}^{*} - {\hat{\boldsymbol{\mu }}}$$.

Assuming that ${\boldsymbol{q}}$ and its speed ${\dot{\boldsymbol{q}}}$ can be measured, the tracking error of the slow subsystem is:

42 $${\boldsymbol{e}} = {\boldsymbol{q}} - {\boldsymbol{q}}_{{\text{d}}}$$

##### Theorem

For the slow subsystem of space robot shown in Eq. (15), by adjusting parameter ${{\varvec{\upbeta}}}_{1}$, $\lambda_{1}$, ${{\varvec{\upbeta}}}_{2}$, $\lambda_{2}$, $c_{1}$, $c_{2}$, the control law Eq. (34) will make the system semi global ultimately uniformly bounded, that is, ${\boldsymbol{e}}$ converges to an arbitrary small neighborhood of zero.

##### Proof

Construct the following Lyapunov function $V$:

43 $$V = V_{1} + V_{2}$$

where $V$ satisfies the initial condition $V\left( 0 \right) \le \rho_{{\text{V}}}$ ($\rho_{{\text{V}}}$ is a positive real number). $V_{1} = \frac{1}{2}{\boldsymbol{s}}_{1}^{{\text{T}}} {\boldsymbol{s}}_{1} + \frac{1}{2}{\boldsymbol{s}}_{2}^{{\text{T}}} {\hat{\overline{\boldsymbol{M}}}}_{ss} {(}{{\varvec{\upupsilon}}}_{1} {)}{\boldsymbol{s}}_{2} + \frac{1}{2}{{\varvec{\upgamma}}}_{2}^{{\text{T}}} {{\varvec{\upgamma}}}_{2}$, $V_{2} = \frac{1}{2}tr\left( {{\tilde{\boldsymbol{W}}}^{{\text{T}}} {{\varvec{\upbeta}}}_{1}^{ - 1} {\tilde{\boldsymbol{W}}}} \right) + \frac{1}{2}tr\left( {{\tilde{\boldsymbol{\mu }}}^{{\text{T}}} {{\varvec{\upbeta}}}_{2}^{ - 1} {\tilde{\boldsymbol{\mu }}}} \right)$.

Find the first derivative of $V$ with regard to time $t$:

44 $$\dot{V} = {\boldsymbol{s}}_{1}^{{\text{T}}} {\dot{\boldsymbol{s}}}_{1} + {\boldsymbol{s}}_{2}^{{\text{T}}} {\hat{\overline{\boldsymbol{M}}}}_{ss} {(}{{\varvec{\upupsilon}}}_{1} {)}{\dot{\boldsymbol{s}}}_{2} + \frac{1}{2}{\boldsymbol{s}}_{2}^{{\text{T}}} {\dot{\hat{\overline{\boldsymbol{M}}}}}_{ss} {(}{{\varvec{\upupsilon}}}_{1} {)}{\boldsymbol{s}}_{2} + {{\varvec{\upgamma}}}_{2}^{{\text{T}}} {\dot{\boldsymbol{\gamma }}}_{2} + tr\left( {{\tilde{\boldsymbol{W}}}^{{\text{T}}} {{\varvec{\upbeta}}}_{1}^{ - 1} {\boldsymbol{\dot{\tilde{W}}}}} \right) + tr\left( {{\tilde{\boldsymbol{\mu }}}^{{\text{T}}} {{\varvec{\upbeta}}}_{2}^{ - 1} {\boldsymbol{\dot{\tilde{\mu }}}}} \right)$$

According to the characteristic that ${\hat{\overline{\boldsymbol{h}}}}_{ss} {(}{{\varvec{\upupsilon}}}_{1} ,{{\varvec{\upupsilon}}}_{2} {)}$ and ${\dot{\hat{\overline{\boldsymbol{M}}}}}_{ss} {(}{{\varvec{\upupsilon}}}_{1} {)}$ satisfy oblique symmetry^16^: $\frac{1}{2}{\boldsymbol{s}}_{2}^{{\text{T}}} {\dot{\hat{\overline{\boldsymbol{M}}}}}_{ss} {(}{{\varvec{\upupsilon}}}_{1} {)}{\boldsymbol{s}}_{2} = {\boldsymbol{s}}_{2}^{{\text{T}}} {\hat{\overline{\boldsymbol{h}}}}_{ss} {(}{{\varvec{\upupsilon}}}_{1} ,{{\varvec{\upupsilon}}}_{2} {)}{\boldsymbol{s}}_{2}$, and Eqs. (39)–(41) are substituted into $\dot{V}_{1}$, it is obtained that:

45 $$\dot{V}_{1} = - c_{1} {\boldsymbol{s}}_{1}^{{\text{T}}} {\boldsymbol{s}}_{1} + {\boldsymbol{s}}_{1}^{{\text{T}}} {\boldsymbol{s}}_{2} + {\boldsymbol{s}}_{1}^{{\text{T}}} {{\varvec{\upgamma}}}_{2} + {\boldsymbol{s}}_{2}^{{\text{T}}} \left[ { - c_{2} {\boldsymbol{s}}_{2} - {\boldsymbol{s}}_{1} + {\tilde{\boldsymbol{W}}}^{{\text{T}}} {\boldsymbol{O}}\left( {\boldsymbol{A}} \right) + {\tilde{\boldsymbol{\mu }}}} \right] - {{\varvec{\upgamma}}}_{2}^{{\text{T}}} \frac{{{{\varvec{\upgamma}}}_{2} }}{{\eta_{2} }} + {{\varvec{\upgamma}}}_{2}^{{\text{T}}} {{\varvec{\Phi}}}\left( {{\boldsymbol{s}}_{1} ,{\boldsymbol{s}}_{2} ,{\boldsymbol{q}}_{{\text{d}}} ,{\dot{\boldsymbol{q}}}_{{\text{d}}} ,{\boldsymbol{\ddot{q}}}_{{\text{d}}} ,{{\varvec{\upgamma}}}_{2} } \right)$$

Substituting the adaptive laws Eqs. (35) and (36) into $\dot{V}_{2}$, we can get:

46 $$\dot{V}_{2} = - tr\left[ {{\tilde{\boldsymbol{W}}}^{{\text{T}}} {\boldsymbol{O}}\left( {\boldsymbol{A}} \right){\boldsymbol{s}}_{2}^{{\text{T}}} } \right] - tr\left( {{\tilde{\boldsymbol{\mu }}}^{{\text{T}}} {\boldsymbol{s}}_{2} } \right) + tr\left( {\lambda_{1} {\tilde{\boldsymbol{W}}}^{{\text{T}}} {{\varvec{\upbeta}}}_{1}^{ - 1} {\hat{\boldsymbol{W}}}} \right) + tr\left( {\lambda_{2} {\tilde{\boldsymbol{\mu }}}^{{\text{T}}} {{\varvec{\upbeta}}}_{2}^{ - 1} {\hat{\boldsymbol{\mu }}}} \right)$$

Using the Inequalities:

47 $$tr\left( {\lambda_{1} {\tilde{\boldsymbol{W}}}^{{\text{T}}} {{\varvec{\upbeta}}}_{1}^{ - 1} {\hat{\boldsymbol{W}}}} \right) \le - \frac{1}{2}tr\left( {\lambda_{1} \widetilde{{\boldsymbol{W}}}^{T} {{\varvec{\upbeta}}}_{1}^{ - 1} \widetilde{{\boldsymbol{W}}}} \right) + \frac{1}{2}tr\left( {\lambda_{1} {\boldsymbol{W}}^{*T} {{\varvec{\upbeta}}}_{1}^{ - 1} {\boldsymbol{W}}^{*} } \right)$$

48 $$tr\left( {\lambda_{2} {\tilde{\boldsymbol{\mu }}}^{{\text{T}}} {{\varvec{\upbeta}}}_{2}^{ - 1} {\hat{\boldsymbol{\mu }}}} \right) \le - \frac{1}{2}tr\left( {\lambda_{2} {\tilde{\boldsymbol{\mu }}}^{{\text{T}}} {{\varvec{\upbeta}}}_{2}^{ - 1} {\tilde{\boldsymbol{\mu }}}^{{\text{T}}} } \right) + \frac{1}{2}tr\left( {\lambda_{2} {{\varvec{\upmu}}}^{{\text{*T}}} {{\varvec{\upbeta}}}_{2}^{ - 1} {{\varvec{\upmu}}}^{*} } \right)$$

Inequality of $\dot{V}$ can be obtained:

49 $$\begin{aligned} \dot{V} & \le - c_{1} {\boldsymbol{s}}_{1}^{{\text{T}}} {\boldsymbol{s}}_{1} - c_{2} {\boldsymbol{s}}_{2}^{{\text{T}}} {\boldsymbol{s}}_{2} + {\boldsymbol{s}}_{1}^{{\text{T}}} {{\varvec{\upgamma}}}_{2} - \frac{1}{2}tr\left( {\lambda_{1} {\tilde{\boldsymbol{W}}}^{T} {{\varvec{\upbeta}}}_{1}^{ - 1} {\tilde{\boldsymbol{W}}}} \right) - \frac{1}{2}tr\left( {\lambda_{2} {\tilde{\boldsymbol{\mu }}}^{{\text{T}}} {{\varvec{\upbeta}}}_{2}^{ - 1} {\tilde{\boldsymbol{\mu }}}^{{\text{T}}} } \right) - {{\varvec{\upgamma}}}_{2}^{{\text{T}}} \frac{{{{\varvec{\upgamma}}}_{2} }}{{\eta_{2} }} \\ &\quad + {{\varvec{\upgamma}}}_{2}^{{\text{T}}} {{\varvec{\Phi}}}\left( {{\boldsymbol{s}}_{1} ,{\boldsymbol{s}}_{2} ,{\boldsymbol{q}}_{{\text{d}}} ,{\dot{\boldsymbol{q}}}_{{\text{d}}} ,{\boldsymbol{\ddot{q}}}_{{\text{d}}} ,{{\varvec{\upgamma}}}_{2} } \right) + \frac{1}{2}tr\left( {\lambda_{1} {\boldsymbol{W}}^{*T} {{\varvec{\upbeta}}}_{1}^{ - 1} {\boldsymbol{W}}^{*} } \right) + \frac{1}{2}tr\left( {\lambda_{2} {{\varvec{\upmu}}}^{{*}{\text{T}}} {{\varvec{\upbeta}}}_{2}^{ - 1} {{\varvec{\upmu}}}^{*} } \right) \end{aligned}$$

According to Young's inequality:

50 $${\boldsymbol{s}}_{1}^{{\text{T}}} {{\varvec{\upgamma}}}_{2} \le \frac{{{\boldsymbol{s}}_{1}^{{\text{T}}} {\boldsymbol{s}}_{1} }}{2} + \frac{{{{\varvec{\upgamma}}}_{2}^{{\text{T}}} {{\varvec{\upgamma}}}_{2} }}{2}$$

51 $${{\varvec{\upgamma}}}_{2}^{{\text{T}}} {{\varvec{\Phi}}}\left( {{\boldsymbol{s}}_{1} ,{\boldsymbol{s}}_{2} ,{\boldsymbol{q}}_{{\text{d}}} ,{\dot{\boldsymbol{q}}}_{{\text{d}}} ,{\boldsymbol{\ddot{q}}}_{{\text{d}}} ,{{\varvec{\upgamma}}}_{2} } \right) \le \frac{{{{\varvec{\upgamma}}}_{2}^{{\text{T}}} {{\varvec{\upgamma}}}_{2} {{\varvec{\Phi}}}^{{\text{T}}} {{\varvec{\Phi}}}}}{2\varsigma } + \frac{\varsigma }{2}$$

where $$\varsigma > 0$$.

$\dot{V}$ is further obtained:

52 $$\begin{aligned} \dot{V} & \le \left( {\frac{1}{2} - c_{1} } \right){\boldsymbol{s}}_{1}^{{\text{T}}} {\boldsymbol{s}}_{1} - c_{2} {\boldsymbol{s}}_{2}^{{\text{T}}} {\boldsymbol{s}}_{2} + {{\varvec{\upgamma}}}_{2}^{{\text{T}}} \left( {\frac{1}{2} - \frac{1}{{\eta_{2} }} + \frac{{{{\varvec{\Phi}}}^{{\text{T}}} {{\varvec{\Phi}}}}}{2\varsigma }} \right){{\varvec{\upgamma}}}_{2} - \frac{1}{2}tr\left( {\lambda_{1} {\tilde{\boldsymbol{W}}}^{T} {{\varvec{\upbeta}}}_{1}^{ - 1} {\tilde{\boldsymbol{W}}}} \right)\\ &\quad - \frac{1}{2}tr\left( {\lambda_{2} {\tilde{\boldsymbol{\mu }}}^{{\text{T}}} {{\varvec{\upbeta}}}_{2}^{ - 1} {\tilde{\boldsymbol{\mu }}}^{{\text{T}}} } \right) + \frac{1}{2}tr\left( {\lambda_{1} {\boldsymbol{W}}^{{*^{T} }} {{\varvec{\upbeta}}}_{1}^{ - 1} {\boldsymbol{W}}^{*} } \right) + \frac{1}{2}tr\left( {\lambda_{2} {{\varvec{\upmu}}}^{{\text{*T}}} {{\varvec{\upbeta}}}_{2}^{ - 1} {{\varvec{\upmu}}}^{*} } \right) + \frac{\varsigma }{2} \end{aligned}$$

According to $\left\| {{\varvec{\Phi}}} \right\| \le \rho_{{\Phi }}$ ($\rho_{{\Phi }}$ is a normal number)^37^, it can be designed that $\frac{1}{{\eta_{2} }} \ge \frac{1}{2} + \frac{{\rho_{{\Phi }}^{2} }}{2\varsigma } + b_{0}$ ($b_{0}$ is a normal number), so that:

53 $$\dot{V} \le - \alpha V + \psi$$

where $$\alpha = \min \left\{ {\left( {2c_{1} - 1} \right),2c_{2} ,2b_{0} ,\lambda_{1} ,\lambda_{2} } \right\}$$, and $$\psi = \frac{\varsigma }{2} + \frac{1}{2}tr\left( {\lambda_{1} {\boldsymbol{W}}^{{*^{T} }} {{\varvec{\upbeta}}}_{1}^{ - 1} {\boldsymbol{W}}^{*} } \right) + \frac{1}{2}tr\left( {\lambda_{2} {{\varvec{\upmu}}}^{{\text{*T}}} {{\varvec{\upbeta}}}_{2}^{ - 1} {{\varvec{\upmu}}}^{*} } \right)$$.

Let $\alpha > {\psi \mathord{\left/ {\vphantom {\psi {\rho_{{\text{V}}} }}} \right. \kern-0pt} {\rho_{{\text{V}}} }}$, then when $V = \rho_{{\text{V}}}$, $\dot{V} \le 0$. It can be seen that $V \le \rho_{{\text{V}}}$ is an invariant set, that is, if $V\left( 0 \right) \le \rho_{{\text{V}}}$, there is $V(t) \le \rho_{{\text{V}}}$ for all $t > 0$.

Solving Eq. (53), we can get:

54 $$0 \le V \le \psi /\alpha + \left[ {V\left( 0 \right) - \psi /\alpha } \right]e^{ - \alpha t}$$

The above formula shows that $V$ is finally bounded by $\psi /\alpha$. Therefore, the system is ultimately uniformly bounded semi globally, and ${\boldsymbol{e}}$ can converge to an arbitrary small neighborhood of zero by adjusting the values of parameters ${{\varvec{\upbeta}}}_{1}$, $\lambda_{1}$, ${{\varvec{\upbeta}}}_{2}$, $\lambda_{2}$, $c_{1}$ and $c_{2}$.

### Linear quadratic controller for fast subsystem

In this work, the linear quadratic regulator is used to control the fast subsystem of the space robot with elastic base and flexible links, so as to actively suppress the vibration of the elastic base and flexible links at the same time.

Writing the fast subsystem Eqs. (17) and (18) into the state equation expression, and making the state variable $${\boldsymbol{P}} = \left[ {\begin{array}{*{20}c} {p_{1} } & {p_{2} } \\ \end{array} } \right]^{{\text{T}}}$$, the Eqs. (17) and (18) are combined as follows:

55 $${\dot{\boldsymbol{P}}} = {\boldsymbol{AP}} + {\boldsymbol{B\tau }}_{f}$$

where $${\boldsymbol{A}} = \left[ {\begin{array}{*{20}c} 0 & {\boldsymbol{I}} \\ { - {\overline{\boldsymbol{N}}}_{ff} {\tilde{\boldsymbol{K}}}} & 0 \\ \end{array} } \right]$$, $${\boldsymbol{B}} = \left[ {\begin{array}{*{20}c} 0 \\ {{\overline{\boldsymbol{N}}}_{fs} } \\ \end{array} } \right]$$.

Equation (55) demonstrates that the fast subsystem is a linear system, and that the system state variable ${\boldsymbol{P}}$ can be adjusted to zero by using the optimal control method, thereby achieving the suppression of base elasticity and flexible links vibration. For a linear system, if the performance index function is defined as the integral of the quadratic function with respect to the state variable and the control variable, the system can obtain the optimal performance by finding the control ${{\varvec{\uptau}}}_{f}$ when the function takes the minimum value.

The function for the linear quadratic optimal control performance index indicator is presented as follows:

56 $$\Upsilon = \frac{1}{2}\int_{0}^{\infty } {{(}{\boldsymbol{P}}^{{\text{T}}} {\boldsymbol{QP}} + {{\varvec{\uptau}}}_{f}^{{\text{T}}} {\boldsymbol{R\tau }}_{f} {\text{)d}}t}$$

where ${\boldsymbol{Q}} \in {\boldsymbol{R}}^{{\left( {\left( {\sum\limits_{i = 1}^{n} {2\kappa_{i} } } \right) + 2} \right) \times \left( {\left( {\sum\limits_{i = 1}^{n} {2\kappa_{i} } } \right) + 2} \right)}}$ is a symmetric weighted matrix with a positive semi-definite, and ${\boldsymbol{R}} \in {\boldsymbol{R}}^{{\left( {n + 1} \right) \times \left( {n + 1} \right)}}$ is a symmetric weighted matrix with a positive definite.

According to the linear quadratic optimal control theory, in order to minimize $\Upsilon$, the control quantity should be created as follows:

57 $${{\varvec{\uptau}}}_{f} = - {\boldsymbol{R}}^{ - 1} {\boldsymbol{B}}^{{\text{T}}} {\boldsymbol{\Xi P}}$$

where ${{\varvec{\Xi}}}$ fulfills the following Riccati algebraic equation.

58 $${\boldsymbol{\Xi A}} + {\boldsymbol{A}}^{{\text{T}}} {{\varvec{\Xi}}} - {\boldsymbol{\Xi BR}}^{ - 1} {\boldsymbol{B}}^{{\text{T}}} {{\varvec{\Xi}}} + {\boldsymbol{Q}} = 0$$

**Figure 2:**
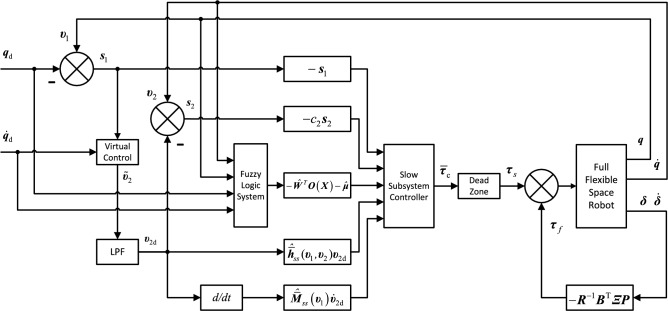
The adaptive fuzzy dynamic surface control scheme for space robot.

Figure 2 shows the block diagram of the control scheme.

## Simulation experiment

**Figure 3:**
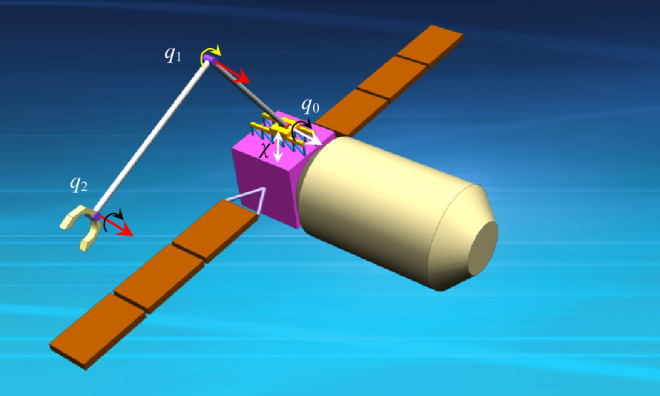
Space Robot System with Elastic base and Flexible Links.

Taking the space robot model system with elastic base and two flexible links shown in Fig. 3 as an example, the numerical simulation experiment is carried out, using a Lenovo Thinkpad X1 with an Intel Core i7-10510U processor, 16 GB memory, Windows 11 and MATLAB. The values of parameters for space robot system and controller are shown in Table 1. The dynamic surface control with adaptive fuzzy approximator (DSC_wAFA) is compared with dynamic surface control without adaptive fuzzy approximator (DSC_woAFA) to evaluate performance of the proposed method.

**Table 1:** The values of parameters.

Parameters	Value
the distance between $O_{1}$ and $O_{0}$	$l_{0} = 1.5{\text{m}}$
The length of $B_{i} \left( {i = 1,2} \right)$ along the $x_{i}$ axis	$l_{1} = 1.5\;{\text{m}}$, $l_{2} = 1.0\;{\text{m}}$
The mass of the base	$m_{0} = 40\;{\text{kg}}$
The moment of inertia about the centre of mass	$J_{0} = 34.17\;{\text{kg}}\;{\text{m}}^{{2}}$
The linear density of flexible arm $B_{i} \left( {i = 1,2} \right)$	$\rho_{1} = 3.5\;{\text{kg/m}}$, $\rho_{2} = 1.1\;{\text{kg/m}}$
The bending stiffness of the section	$\left( {{\text{EI}}} \right)_{1} = 50\;{\text{N}}\;{\text{m}}^{{2}}$, $\left( {{\text{EI}}} \right)_{2} = 50\;{\text{N}}\;{\text{m}}^{{2}}$
Spring stiffness coefficient	$k_{\chi } = 500\;{\text{N/m}}$
The number of fuzzy rules	$\gamma = 5$
Slow change subsystem regulation parameters:	$\lambda_{1} = \lambda_{2} = 0.01$, $\beta_{1} = diag\left\{ {20, \ldots ,20} \right\}$, $\beta_{2} = diag\left\{ {0.01,0.01,0.01} \right\}$, $c_{1} = 10$,$c_{2} = 20$
LQR control parameters	${\boldsymbol{Q}} = diag\left\{ {1, \ldots ,1} \right\}$, ${\boldsymbol{R}} = diag\left\{ {1,1,1} \right\}$
The expected configuration of the attitude angle terminal of the base and the two joints The initial configuration	$${\boldsymbol{q}}_{{\text{d}}} = \left( {\begin{array}{*{20}c} { - \,\uppi /4} & { \,\uppi /2} & {{\uppi /}3} \\ \end{array} } \right)^{{\text{T}}} {\text{rad}}$$ $${\boldsymbol{q}}{(0)} = \left( {\begin{array}{*{20}c} { -\uppi {/4} - {0}{\text{.1}}} & {\uppi {/2 + 0}{\text{.2}}} & {\uppi {/3 + 0}{\text{.3}}} \\ \end{array} } \right)^{{\text{T}}} {\text{rad}}$$
The initial displacement of the base spring	$\chi \left( 0 \right) = 0$
The number of truncated terms	$\kappa_{1} = \kappa_{2} { = }2$
Set the track tracking process simulation time	$t = 20{\text{s}}$

**Figure 4:**
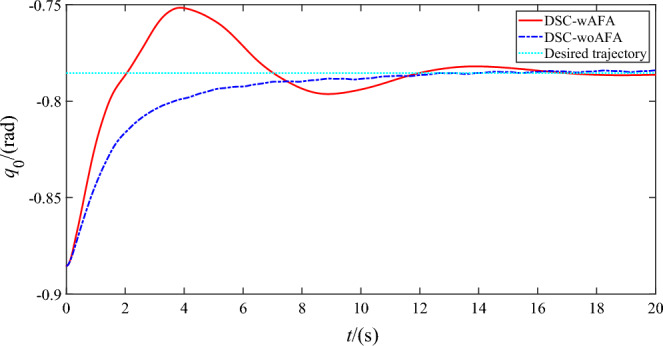
Trajectory tracking of the base’s attitude.

**Figure 5:**
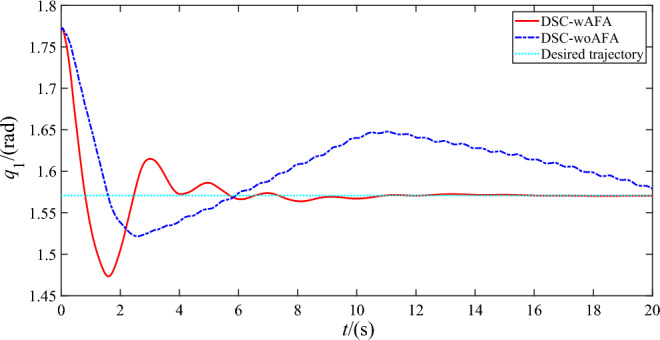
Trajectory tracking of the joint angle 1.

**Figure 6:**
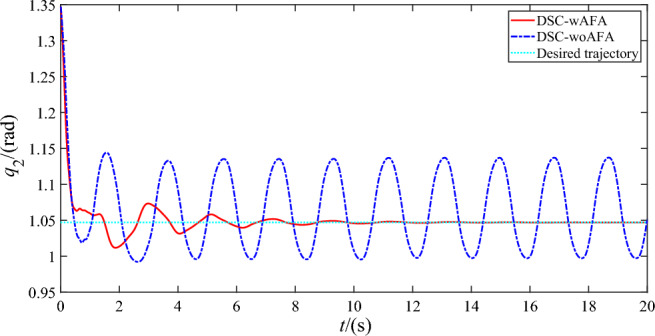
Trajectory tracking of the joint angle 2.

Figures 4, 5 and 6 shows the trajectory tracking of rigid motion of the base ‘s attitude and two joint angles of the space robot system using DSC-wAFA and DSC-woAFA in the case of dead zone in joint input torque. When the results of the DSC-wAFA is compared with and DSC-woAFA, it is shown that the actual trajectory of the base ‘s attitude and two joint angles can track the desired trajectory effectively with proposed control method and there is obvious tracking error of two joint angles in the DSC-woAFA system.

**Figure 7:**
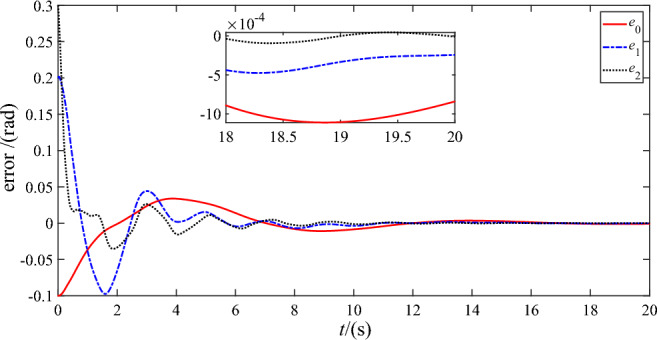
Trajectory tracking error of DSC-wAFA.

**Figure 8:**
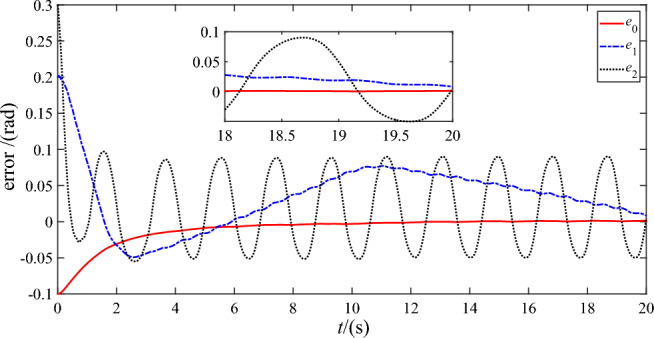
Trajectory tracking error of DSC-woAFA.

Figures 7 and 8 show the trajectory tracking errors of the base ‘s attitude and two joint angles utilizing DSC-wAFA and DSC-woAFA, respectively. It can be seen from the partial enlarged view of Fig. 7 that, after about $18{\text{s}}$, the steady-state errors of base’s attitude, joint angle 1 and joint angle 2 are within $1.1 \times 10^{ - 3} {\text{rad}}$, $5 \times 10^{ - 4} {\text{rad}}$ and $1.1 \times 10^{ - 4} \;{\text{rad}}$ respectively. Therefore, the proposed anti-deadzone controller gives satisfactory performance. Through comparison, it can be seen that when the dead zone adaptive fuzzy approximator is turned off, the tracking error of base’s attitude, joint angle 1 and joint angle 2 are within $1.4 \times 10^{ - 3} \;{\text{rad}}$, $0.03\;{\text{rad}}$ and $0.1\;{\text{rad}}$ respectively, and the tracking error of both joints cannot converge due to damage of the deadzone in joint input torque of two links.

**Figure 9:**
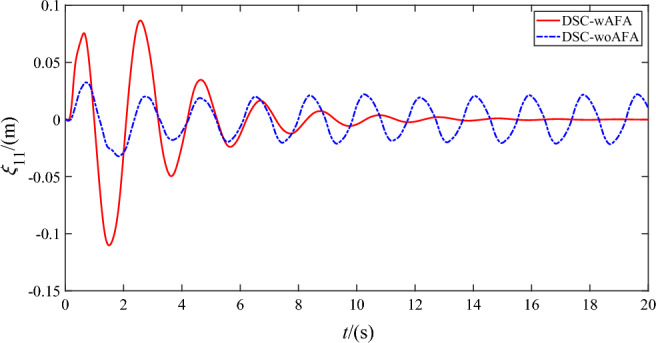
The first mode of flexible link $B_{1}$.

**Figure 10:**
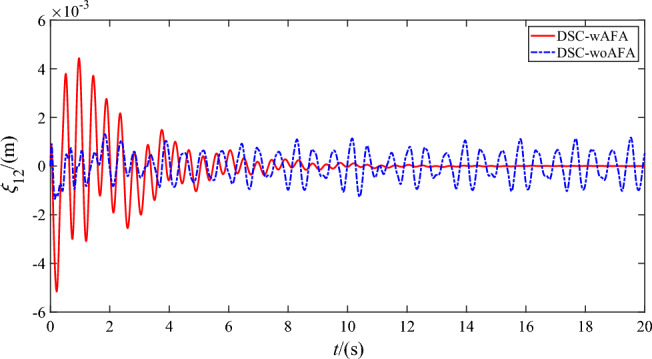
The second mode of flexible link $B_{1}$.

**Figure 11:**
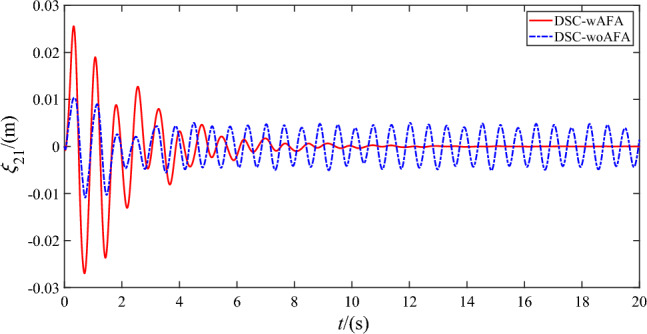
The first mode of flexible link $B_{2}$.

**Figure 12:**
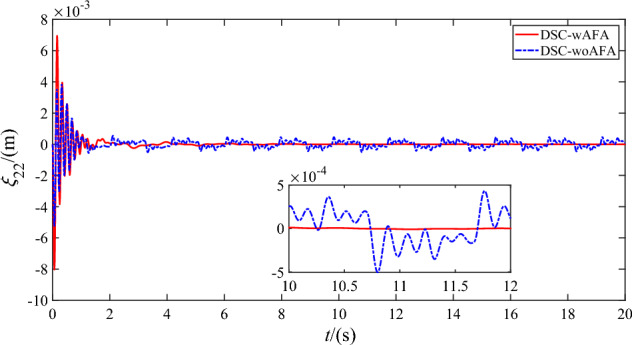
The second mode of flexible link $B_{2}$.

**Figure 13:**
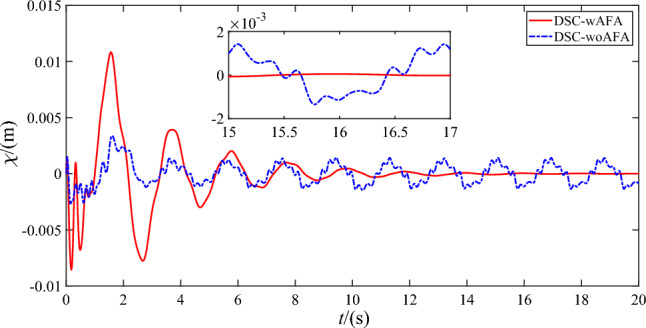
Elastic displacement of the base.

Figures 9, 10, 11 and 12 shows the flexible modes of two links of the space robot system using DSC-wAFA and DSC-woAFA in the case of dead zone. The first mode of $B_{1}$ attenuates from $0.11{\text{m}}$ at $1.5{\text{s}}$ to zero in the DSC-wAFA system, but vibrates between $\pm 0.02{\text{m}}$ after $4{\text{s}}$ in the DSC-woAFA system. The second mode of $B_{1}$ attenuates from $0.005{\text{m}}$ at $0.2{\text{s}}$ to zero in the DSC-wAFA system, but vibrates between $\pm 0.012{\text{m}}$ after $4{\text{s}}$ in the DSC-woAFA system. The first mode of $B_{2}$ attenuates from $0.027{\text{m}}$ at $0.7{\text{s}}$ to zero in the DSC-wAFA system, but vibrates between $\pm 0.005{\text{m}}$ after $4{\text{s}}$ in the DSC-woAFA system. The second mode of $B_{2}$ attenuates from $0.008{\text{m}}$ at $0.06{\text{s}}$ to zero in the DSC-wAFA system, but vibrates between $\pm 0.0005{\text{m}}$ after $4{\text{s}}$ in the DSC-woAFA system. Figure 13 shows the elastic vibration of the base of the space robot system. The elastic displacement is $0.011{\text{m}}$ at $1.58{\text{s}}$ and attenuates to zero after $15{\text{s}}$ in the DSC-wAFA system, but vibrates between $\pm 0.0015{\text{m}}$ after $4{\text{s}}$ in the DSC-woAFA system. It is demonstrated that, when the rigid motion trajectory is stable in the DSC-wAFA system, the elastic oscillation of the base and the vibration of the two flexible links are suppressed by using linear quadratic controller. Thus, the effectiveness of the vibration suppression scheme is verified.

**Figure 14:**
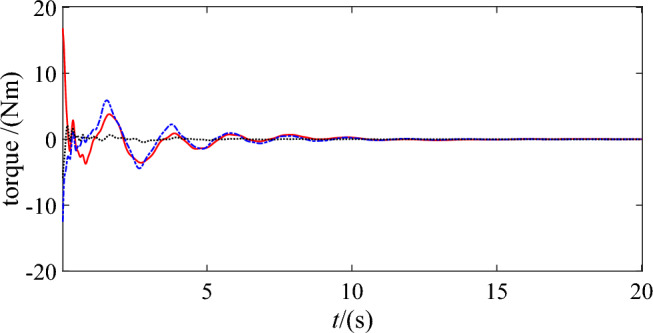
Control torque of DSC-wAFA.

**Figure 15:**
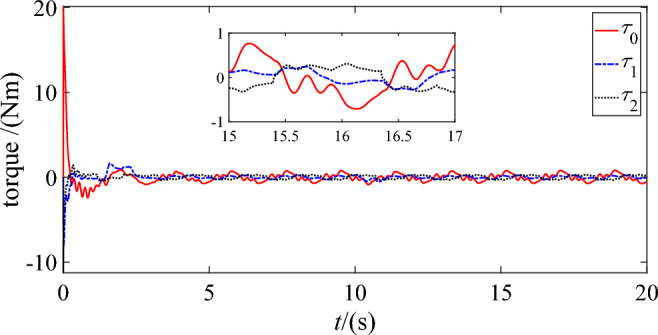
Control torque of DSC-woAFA.

Figures 14 and 15 show the control torques of the base ‘s actuator and two joints utilizing DSC-wAFA and DSC-woAFA respectively. The control torques converge to zero when the DSC-wAFA system is stable, but are oscillating in the DSC-woAFA system.

From the simulation results of the proposed controller, it can be seen that the base’s attitude and the two joint angles can track the desired trajectory of rigid motion, and the vibration of elastic base and flexible links are damped out at the same time. Therefore, the proposed control scheme can tackle the effect of dead-zone and dual vibration effectively, with high tracking accuracy and satisfactory performance.

## Conclusions

1. Considering the multiple coupling between the elastic base and the flexible links, the dynamic equations of the space robot with elastic base and flexible links are derived by integrating the momentum conservation relation of the system, the second kind of Lagrange equation and the assumed mode method.
2. Based on the singular perturbation method, the system is decomposed into slow and fast subsystems, which describe the rigid motion, the base elasticity, and the flexible vibrations of links respectively. For the slow subsystem, a dynamic surface controller with adaptive fuzzy approximator is designed when there is dead zone in joint input torque. For the fast subsystem, the optimal quadratic controller is adopted. The combined control scheme can not only overcome the adverse effects of the dead zone on the system, ensure that the space robot system can track the desired trajectory of the rigid motion, but also actively suppress the vibration of the base elasticity and the flexible links at the same time, overcome the impact of unknown inertia parameters, and meet the control requirements.
3. This paper presents a theoretical exploration and preparatory study. When the hardware conditions and relevant assumptions fulfill the requirements, the proposed control scheme can effectively guide and be applied to practical systems. Although the focus of this research is on a planar space robot, its applicability can be extended to general space robot systems with multiple degrees of freedom.

## Data Availability

The data presented in this study are available on request from the corresponding author.
